# The correlation between serum apolipoprotein B/apolipoprotein A1 ratio and brain necrosis in patients underwent radiotherapy for nasopharyngeal carcinoma

**DOI:** 10.1002/brb3.1554

**Published:** 2020-02-03

**Authors:** Honghong Li, Dong Zheng, Fukang Xie, Xiaolong Huang, Xiaohuang Zhuo, Jinpeng Lin, Yi Li, Yamei Tang

**Affiliations:** ^1^ Department of Neurology Sun Yat‐sen Memorial Hospital Sun Yat‐sen University Guangzhou China; ^2^ Department of Neurology The Affiliated Brain Hospital of Guangzhou Medical University (Guangzhou Huiai Hospital) (Guangdong Engineering Technology Research Center for Translational Medicine of Mental Disorders) Guangzhou China; ^3^ Histology and Embryology Department of Zhongshan School of Medicine Sun Yat‐sen University Guangzhou China; ^4^ Department of Intensive Care Unit The First Affiliated Hospital of Xiamen University Xiamen China; ^5^ Guangdong Provincial Key Laboratory of Malignant Tumor Epigenetics and Gene Regulation Sun Yat‐Sen Memorial Hospital Sun Yat‐Sen University Guangzhou China; ^6^ Guangdong Province Key Laboratory of Brain Function and Disease Zhongshan School of Medicine Sun Yat‐Sen University Guangzhou China

**Keywords:** apolipoprotein B/apolipoprotein A1 ratio, nasopharyngeal carcinoma, quality of life, radiation‐induced brain necrosis

## Abstract

**Introduction:**

The apolipoprotein B/apolipoprotein A1 (ApoB/ApoA1) ratio is recognized as a clinical indicator of cardiovascular disease and ischemic cerebral disease. Cerebrovascular dysfunction is also involved in head and neck radiotherapy. The aim of this study was to investigate the correlation between ApoB/ApoA1 ratio and the severity of radiation‐induced brain necrosis (RN) in patients who underwent radiotherapy after nasopharyngeal carcinoma (NPC).

**Methods:**

In this retrospective study, 191 NPC patients diagnosed with RN were evaluated. Clinical characteristics, serum lipid, apolipoproteins, and brain magnetic resonance imaging findings were collected. Serum lipid and apolipoproteins were quantified using standard diagnostic assays, and the quality of life (QOL) was assessed by the World Health Organization quality of life abbreviated instrument (WHOQOL‐BREF).

**Results:**

ApoB/ApoA1 ratio was positively correlated with lesion volume (*r* = .18, *p* = .03) and negatively correlated with WHOQOL‐BREF scores (*r* = −.28, *p* < .01). The ApoB/ApoA1 ratio and intensity‐modulated radiation therapy (IMRT) were independent risk factor of RN volume. Moreover, ApoB/ApoA1 ratio was significantly negatively correlated with physical health (*r* = −.29, *p* < .01), psychological (*r* = −.27, *p* < .01), social relationships (*r* = −.17, *p* = .02), and environment (*r* = −.27, *p* < .01) domains of WHOQOL‐BREF.

**Conclusions:**

Serum ApoB/ApoA1 ratio is positively correlated with RN volume, which indicated serum ApoB/ApoA1 ratio as an independent risk factor for lesion volume in patients with RN after radiotherapy for NPC, suggesting a bright intervention target in RN treatment.

## Graphical abstract

Radiotherapy causes burdensome side effects that seriously affected the quality of life (QOL). Apolipoprotein B/apolipoprotein A1 (ApoB/ApoA1) ratio was positively correlated with radiotherapy induce brain necrosis (RN) volume and negatively correlated with QOL. ApoB/ApoA1 ratio was an independent risk factor of RN volume, suggesting a bright intervention target in RN treatment.

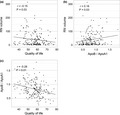

## 1 INTRODUCTION

Nasopharyngeal carcinoma (NPC) is known as a geographical disease with a high incidence in Southern China and Southeast Asia (Zhang et al., 2015). Radiotherapy with or without concurrent chemotherapy is one of the most successful and widely used treatments for NPC (Chan, 2010). Although it has contributed to increased survival rates, it frequently causes burdensome side effects, such as cognitive impairment (Shen et al., 2016), epilepsy (Rong, Yin, Wang, Zhang, & Peng, 2017), and even coma from a brain herniation (Van Dycke et al., 2013), which seriously affected the quality of life (QOL; Zhou et al., 2018).

Among traditional cardiovascular risk factors, apolipoprotein B/apolipoprotein A1 (ApoB/ApoA1) ratio is recognized to have the strongest predictive value for ischemic stroke (Kostapanos et al., 2010). A meta‐analysis suggests that increased ApoB/ApoA1 ratio was risk factor for a first cerebral ischemic (Dong et al., 2015). Jong‐Ho Park also suggested that a higher ApoB/ApoA1 ratio is a predictor of intracranial atherosclerotic stenosis in Asian patients with stroke (Park, Hong, Lee, Kim, & Song, 2013). Apolipoprotein A1 (ApoA1) is the major apolipoprotein in high‐density lipoprotein cholesterol (HDL‐C), which initiates reverse cholesterol transport from blood vessels to the liver (Nissen et al., 2003), and it also has the effect of anti‐inflammation and antioxidation (Walldius & Jungner, 2006). In contrast, apolipoprotein B (ApoB) produces various proinflammatory factors and promotes atherogenesis in the atrial wall (Sniderman & Faraj, 2007). Accordingly, ApoB/ApoA1 ratio has been considered as a marker of the balance between proatherogenic and antiatherogenic lipoprotein particles (Milionis, Liberopoulos, et al., 2005). These suggested that ApoB/ApoA1 ratio plays a crucial role in vascular function. Previous study suggested that radiation can cause middle cerebral artery stenosis in patients with NPC after radiotherapy (Zhou et al., 2016). A high prevalence of cerebral microbleeds (CMBs) was found in patients after cranial irradiation, which progressively increasing with each year from time of radiotherapy (Roongpiboonsopit et al., 2017). Additionally, in our previous study, we also demonstrated that cognitive impairment was resulted from (CMBs) in patients with radiation‐induced brain necrosis (RN) (Shen et al., 2016). Above all, cerebrovascular dysfunction is widely involved in RN. These results raise the issue of whether ApoB/ApoA1 ratio correlated with the RN. So, we design this study to explore the correlation between the ApoB/ApoA1 ratio and the RN after radiotherapy for NPC patients.

## 2 METHODS

This retrospective study was approved by an authorized human research review board in our hospital.

### 2.1 Study population

#### 2.1.1 Patients

In this retrospective study, we reviewed the charts of patients who were diagnosed with RN after radiotherapy for NPC between February 2014 and February 2017 in our hospital. Eligibility criteria for inclusion were described as follows: (a) Patients aged ≥18 years; (b) radiographic evidence showed RN without tumor recurrence or metastases. Patients who had been on statin or fibrate were excluded (Walldius & Jungner, 2004). A total of 214 NPC patients with NPC who received radiotherapy were screened, and 23 patients were excluded including 3 of NPC relapse, 18 of statin using, and 2 of fibrate using. Thus, 191 patients met these criteria and were included in our analysis.

#### 2.1.2 Data collection

Demographics, clinical, and laboratory data were collected, including age, sex, TNM stage (American Joint Committee on Cancer [AJCC] 8th edition), total radiation dose for nasopharyngeal/neck, radiation approaches (intensity‐modulated radiotherapy [IMRT] vs. conventional), chemotherapy or not, total cholesterol, triglycerides, low‐density lipoprotein cholesterol (LDL‐C) levels, HDL‐C levels, ApoA1 levels, and ApoB levels. The RN edema volume of the patients was detected by using T2‐weighted fluid‐attenuated inversion recovery (FLAIR; Li et al., 2018) and was independently assessed by a radiologists who were blinded to the diagnosis and treatment. For volume of lesion measurement, radiologists identified the outline of the lesion manually and semiautomatically, and the total RN volume was estimated with Volume Viewer 2 software (GE Healthcare, AW Suite 2.0 6.5.1. z) on T2‐weighted FLAIR images (Li et al., 2018). The T2‐weighted FLAIR image was performed when RN was diagnosed for the first time in our hospital.

#### 2.1.3 Blood test

All these blood tests were performed using commercially available standardized methods when RN was diagnosed for the first time in our hospital. Blood samples were drawn in the morning after an overnight fast for 12 hr (Milionis, Rizos, et al., 2005). All biochemical levels were measured in the clinical laboratory of the hospital by use of a Hitachi 7600‐020 automatic biochemical analyzer (Beckman CX‐7 Biochemical Autoanalyser). Serum levels of cholesterol, triglycerides, HDL, and LDL were assayed by an enzymatic technique (Park, Hong, Lee, Lee, & Kim, 2011). The ApoA1 and ApoB levels were measured with ApoA1 and ApoB kits separately by immunoturbidimetry assay (Beckman Coulter, Inc.).

#### 2.1.4 Assessment of QOL

Quality of life was assessed using the World Health Organization Quality of Life Abbreviated Instrument (WHOQOL‐BREF), which comprises 26 items and measure the following broad domains: physical health, psychological health, social relationships, and environment (Skevington, Lotfy, & O'Connell, 2004; Tang, Luo, Rong, Shi, & Peng, 2012). Each item was scored from 1 to 5 points, and a higher score indicates a better QOL. The 4–20 scale was used to transform domain scores for each domain in this study. The domain scores were calculated by multiplying the average scores of all items in the domain by a factor of 4. Therefore, each domain score would have the same range (from 4 to 20). This questionnaire is used by a large number of research groups in cancer clinical trials (Bouya et al., 2018; Vranceanu et al., 2016). The WHOQOL‐BREF was measured on every patient when RN was diagnosed for the first time in our hospital.

### 2.2 Statistical analysis

Statistical analyses were performed using R for Windows (version 3.4.2, http://www.r-project.org/). The data were presented as mean (*SD*), median (IQR), or number (%). Linear regression model was used for the univariate and multivariate analyses of QOL and RN volume with demographic characteristics, lipid parameters, and treatment‐related characteristics. A final model selection was performed by backward stepwise regression with Akaike information criterion (AIC). The correlation between ApoB/ApoA1 ratio and QOL was determined by multivariate linear regression modeling after adjusting for chemotherapy. Pearson's correlation test was used to assess the linear dependence among ApoB/ApoA1 ratio, WHOQOL‐BREF, and RN volume. All statistical tests were two‐sided, and *p* values of less than .05 were considered to be statistically significant.

## 3 RESULTS

### 3.1 Clinical characteristics

In total, 191 patients with a mean age of 50 ± 8 years were included in the study. The demographic data and clinical characteristics are presented in Table 1. In these 191 patients, the median WHOQOL‐BREF score was 56.60 ± 8.48, and the median RN volume was 18.9 ± 51.05 mm^3^.

**Table 1:** Baseline characteristics of study population

Demographic and clinical characteristics		NPC‐associated characteristics	
Age, years	50.00 [46.00, 56.00]	T	
Sex		T1	10 (5.2%)
Men	146 (76.4%)	T2	31 (16.2%)
Women	45 (23.6%)	T3	97 (50.8%)
Diabetes	3 (1.6%)	T4	53 (27.8%)
Hypertension	35 (18.3%)	N	
Cholesterol, mmol/L	5.11 [4.44, 5.67]	N0	33 (17.3%)
Triglycerid, mmol/L	0.98 [0.66, 1.40]	N1	94 (49.2%)
HDL‐C, mmol/L	1.28 [1.11, 1.52]	N2	55 (28.8%)
LDL‐C, mmol/L	3.26 [2.66, 3.73]	N3	9 (4.7%)
ApoliA1, g/L	1.16 [1.04, 1.29]	Stage	
ApoliB, g/L	0.94 [0.79, 1.07]	1 + 2	22 (11.5%)
ApoB/ApoA1	0.80 [0.66, 1.00]	3	113 (59.2%)
DAR, year	6.30 [4.13, 9.25]	4	56 (29.3%)
DRNR, year	3.97 [2.88, 6.09]		
DARN, year	1.30 [0.10, 2.80]		
QOL	56.60 (8.48)		
RN volume, mm^3^	18.90 [5.19, 56.25]		

Radiation‐related characteristics		Chemotherapy	
Prescribed radiation dose of the NP, Gy	70.00 [70.00, 72.00]	Platinum	148 (77.5%)
Prescribed radiation dose of the neck, Gy	62.00 [60.00, 66.00]	Fluorouracil	77 (40.3%)
Radiation approaches		Docetaxel	31 (16.2%)
Traditional	132 (69.1%)	Bleomycin	4 (2.1%)
IMRT	59 (30.9%)		

Note

The normal range for laboratory values: TC, 2.9–6.0 mmol/L; TG, 0.31–2.3 mmol/L; HDL‐C, 0.8–1.96 mmol/L; LDL‐C, 1.3–3.6 mmol/L; ApolA1, 1.0–1.6 g/L; ApolB, 0.5–1.10 g/L. Data are presented as mean (*SD*), median (IQR), or *n* (%).

Abbreviations: ApolA1, apolipoprotein A1; ApolB, apolipoprotein B; DAR, duration between RT and assessment; DARN, duration between assessment and radiotherapy‐induced necrosis; DRNR, duration between radiotherapy‐induced necrosis and radiotherapy; Gy, Gray; HDL‐C, high‐density lipoprotein cholesterol; IMRT, intensity‐modulated radiation therapy; LDL‐C, low‐density lipoprotein cholesterol; NP, nasopharyngeal; NPC, nasopharyngeal carcinoma; QOL, quality of life; RN, radiation‐induced brain necrosis.

### 3.2 Correlation among ApoB/ApoA1 ratio, RN volume, and WHOQOL‐BREF score

**Figure 1:**
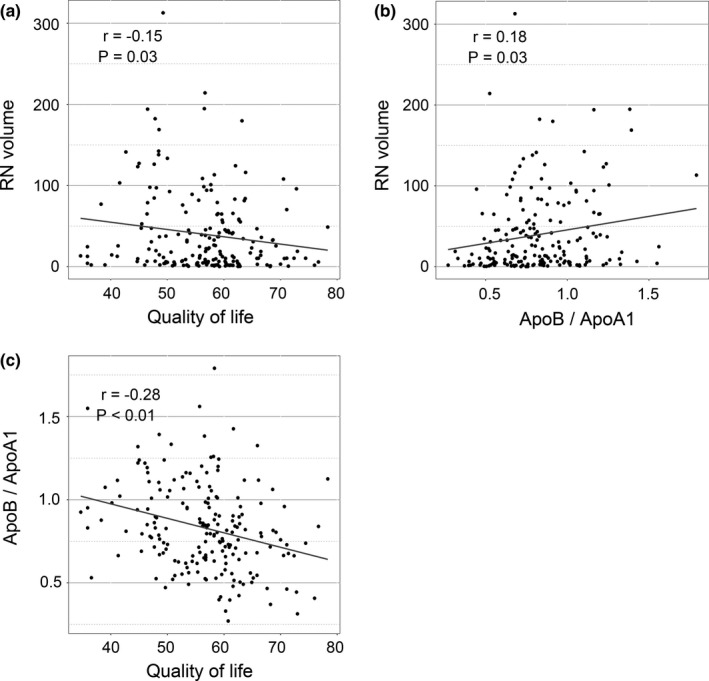
Correlations among apolipoprotein B/apolipoprotein A1 (ApoB/ApoA1) ratio, radiation‐induced brain necrosis (RN) volume, and quality of life (QOL) in patients with RN. (a) Correlation between RN volume and QOL (*r* = −.15, *p* = .03). (b) Correlation between ApoB/ApoA1 ratio and RN volume (*r* = .18, *p* = .03). (c) Correlation between ApoB/ApoA1 ratio and QOL (*r* = −.28, *p* < .01)

**Figure 2:**
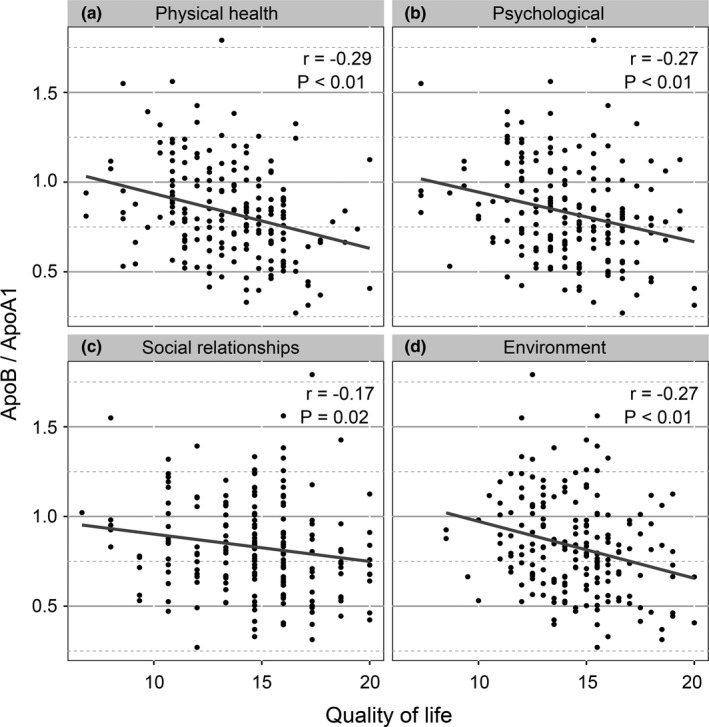
Correlations between apolipoprotein B/apolipoprotein A1 (ApoB/ApoA1) ratio and four domains of World Health Organization quality of life abbreviated instrument (WHOQOL‐BREF) in patients with radiation‐induced brain necrosis (RN). (a) Correlation between ApoB/ApoA1 ratio and the score for the physical health domain of WHOQOL‐BREF (*r* = −.29, *p* < .01). (b) Correlation between ApoB/ApoA1 ratio and the score for the psychological health domain of WHOQOL‐BREF (*r* = −.27, *p* < .01). (c) Correlation between ApoB/ApoA1 ratio and the score for the social relationships domain of WHOQOL‐BREF (*r* = −.17, *p* = .02). (d) Correlation between ApoB/ApoA1 ratio and the score for the environment domain of WHOQOL‐BREF (*r* = −.27, *p* < .01)

Firstly, ApoB/ApoA1 ratio was significantly positively correlated with RN volume (*r* = .18, *p* = .03) and was negatively correlated with total WHOQOL‐BREF scores (*r* = −.28, *p* < .01) (Figure 1). RN volume was significantly negatively correlated with total WHOQOL‐BREF scores (*r* = −.15, *p* = .03) (Figure 1). Then, we investigated the correlation between ApoB/ApoA1 ratio and four domains of WHOQOL‐BREF. As shown in Figure 2, significant negative correlations between ApoB/ApoA1 ratio and physical health (*r* = −.29, *p* < .01), psychological (*r* = −.27, *p* < .01), social relationships (*r* = −.17, *p* = .02), and environment (*r* = −.27, *p* < .01) domains of WHOQOL‐BREF were observed.

**Figure 3:**
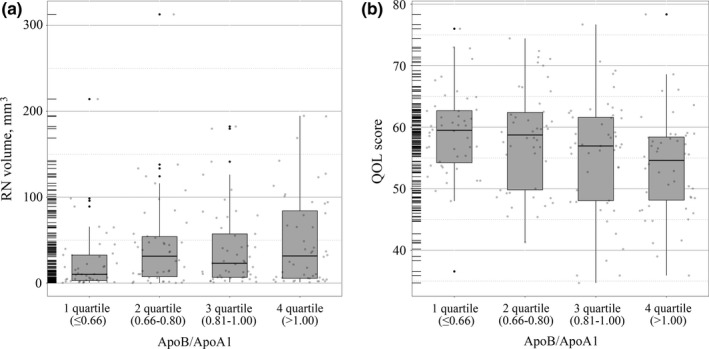
Relationships between apolipoprotein B/apolipoprotein A1 (ApoB/ApoA1) ratio quartiles and the radiation‐induced brain necrosis (RN) volume and quality of life (QOL). Patients with higher quartiles of the ApoB/ApoA1 ratio had a larger RN volume (a, *p* < .05) and a lower QOL scores (b, *p* < .05)

In addition, we analyzed again by changing continuous variables of ApoB/ApoA1 ratio into categorical variables. The results showed that patients with higher quartiles of the ApoB/ApoA1 ratio had a larger RN volume (Figure 3a, *p* < .05) and a lower QOL scores (Figure 3b, *p* < .05).

### 3.3 Risk factors of RN volume and QOL

Finally, we analyzed the correlation of baseline clinical features, lipid profiles, and treatment‐related characteristics with RN volume and QOL. We found that HDL, ApoB/ApoA1 ratio, DARN, and IMRT were significantly correlated with RN volume (Table 2). No evidence for any correlation between RN volume and sex, age, cholesterol, triglycerides, LDL, dose of radiation, and chemotherapy approaches. Then, a final model selection was performed by backward stepwise regression with AIC, which showed that higher ApoB/ApoA1 ratio and IMRT were significant independent risk factor of RN volume (Table 2). Additionally, higher ApoB/ApoA1 ratio, T3 stage, and N1 stage were significant independent risk factors of QOL (Table 3).

**Table 2:** Univariate and multivariate linear regression analysis on factors associated with RN

	Univariate analysis		Multivariate analysis	
	Coefficient	*p* Value	Coefficient	*p* Value
Clinical and lipids characteristics				
Age, years				
≤40	Ref			
41–50	−1.89	.88		
51–60	8.41	.50		
>60	0.29	.98		
Sex				
Men	Ref			
Women	−8.70	.30		
Hypertension	2.69	.77		
Cholesterol, mmol/L	−3.05	.32		
Triglycerid, mmol/L	5.13	.27		
HDL, mmol/L	−25.97	.01		
LDL, mmol/L	0.04	.99		
ApoliA, g/L				
<Median (1.16)	Ref			
≥Median (1.16)	−6.52	.37		
ApoliB, g/L				
<Median (0.94)	Ref			
≥Median (0.94)	9.58	.18		
ApoB/ApoA1, quartile				
1 (≤0.66)	Ref		Ref	
2 (0.66–0.80)	19.74	.05	25.50	.01
3 (0.81–1.00)	15.27	.13	19.18	.05
4 (>1.00)	24.38	.02	30.69	.003
DAR, years	−0.44	.59		
DRNR, years	−1.35	.13		
DARN, years	2.92	.08		
Tumor and treatment characteristics				
Prescribed radiation dose of the NP, Gy				
≤70	Ref			
>70	3.51	.63		
Prescribed radiation dose of the neck, Gy				
≤60	Ref			
>60	−10.98	.13		
Radiation approaches				
Traditional	Ref		Ref	
IMRT	−15.43	.04	−21.63	.01
Chemotherapy				
Platinum	−2.98	.73		
Fluorouracil	−1.21	.87		
Docetaxel	3.03	.76		
T				
1 + 2	Ref			
3	8.09	.38		
4	12.75	.22		
N				
0	Ref			
1	−3.29	.74		
2 + 3	−1.56	.88		

Note

The final multivariate linear regression model selection was performed by backward stepwise regression with Akaike information criterion.

Abbreviations: ApolA1, apolipoprotein A1; ApolB, apolipoprotein B; Gy, Gray; IMRT, intensity‐modulated radiation therapy; RN, radiation‐induced brain necrosis.

**Table 3:** Univariate and multivariate linear regression analysis on factors associated with QOL

	Univariate analysis		Multivariate analysis	
	Coefficient	*p* Value	Coefficient	*p* Value
Clinical and lipids characteristics				
Age, years				
≤40	Ref			
41–50	3.93	.06		
51–60	3.22	.12		
>60	−0.39	.88		
Sex				
Men	Ref			
Women	1.64	.26		
Hypertension	−1.03	.52		
Cholesterol, mmol/L	−0.80	.13		
Triglycerid, mmol/L	0.00	1.00		
HDL, mmol/L	4.30	.01		
LDL, mmol/L	−1.25	.06		
ApoliA, g/L				
<Median (1.16)	Ref			
≥Median (1.16)	0.55	.66		
ApoliB, g/L				
<Median (0.94)	Ref			
≥Median (0.94)	−4.03	<.001		
ApoB/ApoA1, quartile				
1 (≤0.66)	Ref		Ref	
2 (0.66–0.80)	−1.00	.56	−1.37	.42
3 (0.81–1.00)	−3.64	.03	−4.13	.01
4 (>1.00)	−5.40	.002	−5.12	.003
DAR, years	0.01	.96		
DRNR, years	−0.06	.72		
DARN, years	0.16	.57		
Tumor and treatment characteristics				
Prescribed radiation dose of the NP, Gy				
≤70	Ref			
>70	−0.14	.91		
Prescribed radiation dose of the neck, Gy				
≤60	Ref			
>60	0.43	.73		
Radiation approaches				
Traditional	Ref			
IMRT	−2.15	.11		
Chemotherapy				
Platinum	−2.40	.10		
Fluorouracil	−2.48	.048		
Docetaxel	2.22	.18		
T				
1 + 2	Ref		Ref	
3	3.13	.047	3.61	.02
4	0.67	.70	1.38	.42
N				
0	Ref		Ref	
1	−4.32	.01	−3.98	.02
2 + 3	−3.58	.048	−2.33	.19

Note

The final multivariate linear regression model selection was performed by backward stepwise regression with Akaike information criterion.

Abbreviations: ApolA1, apolipoprotein A1; ApolB, apolipoprotein B; Gy, Gray; IMRT, intensity‐modulated radiation therapy; QOL, quality of life.

## 4 DISCUSSION

Several studies have indicated an correlation of the ApoB/ApoA1 ratio and the risk of stroke (Dong et al., 2015; Kostapanos et al., 2010), intracranial and extracranial carotid stenosis (Park et al., 2011, 2013). But no prior study has demonstrated a correlation between the ApoB/ApoA1 ratio and RN in patients after radiotherapy for NPC. This study suggests that elevated serum ApoB/ApoA1 ratio was positively correlated with RN volume and negatively associated with WHOQOL‐BREF scores, which indicated serum ApoB/ApoA1 ratio as an independent risk factor for RN volume and QOL.

How does ApoB/ApoA1 ratio affect the RN? Accumulating evidence suggests that elevated ApoB/ApoA1 ratio enhanced atherosclerotic burden (Sniderman et al., 2003). It was repeatedly demonstrated that elevated ApoB/ApoA1 ratio played a risk role in ischemic stroke (Dorresteijn et al., 2002; Gujral et al., 2016). Weintraub, Jones, and Manka (2010) reported that radiotherapy may damage endothelial cells by radiation impairment on DNA and subcellular structures. Also, Halle et al. (2010) demonstrated that radiation leads to radiation‐induced vascular disease by up‐regulation of nuclear factor kappa‐light‐chain‐enhancer of activated B cells (NF‐κB), which contribute to the pathology by inducing proinflammatory genes. Inflammatory injury is a major mechanism underlying late radiation toxicity in the central nervous system. The ApoA1 level refers to a marker of antioxidant and anti‐inflammatory properties (Walldius & Jungner, 2006). While, the ApoB is a marker of oxidative and atherogenic properties (Dong et al., 2015). The elevated ApoB/ApoA1 ratio mirrors the unbalance of proinflammation and anti‐inflammation, which might enhance the RN. Therefore, increased ApoB/ApoA1 ratio exacerbating the atherosclerosis and inflammation of intracranial vascular might explain our findings of a correlation of the ApoB/ApoA1 ratio with RN volume. Additionally, Clement (2014) suggested that cranial irradiation in humans implied a recognized increased risk for neurovascular atherosclerosis, which support our findings. Furthermore, IMRT was also a risk factor of RN volume, which may be related to the better normal tissue sparing of IMRT.

ApoB/ApoA1 ratio was negatively correlated with WHOQOL‐BREF, which may be attributed to the increase in lesion volume. The result that ApoB/ApoA1 ratio was positively correlated with RN volume, and RN volume was negatively correlated with WHOQOL‐BREF supports this claim. As the elevated ApoB/ApoA1 ratio may lead to large RN volume, the increased lesion volume may contribute to cognition impairment (Xu et al., 2018). Cognition impairment is one of the most common complaints and poses a critical impact on QOL in these patients. In addition, patients with larger RN volume are more likely to suffer from epilepsy, thereby lowing the QOL. Additionally, elevated ApoB/ApoA1 ratio was an independent risk factor for QOL. While, a higher LDL‐C level failed to indicate an increased risk of poor QOL. These findings conform to recent studies that suggested the ApoB/ApoA1 ratio is a good predictor of intracranial carotid stenosis and cardiovascular disease than other traditional cholesterol measures (Park et al., 2011; Yusuf et al., 2004).

Therefore, therapeutic approaches aimed to decrease ApoB/ApoA1 ratio as a residual risk control may be potentially beneficial in decreasing the RN volume and improving the QOL. WHOQOL‐BREF scores have been considered to be a prognostic factor for cancer patients, such as for head and neck cancer (Karvonen‐Gutierrez et al., 2008; Siddiqui et al., 2008). The clinical implication of our findings lies in the potential of ApoB/ApoA1 ratio as a reliable index to distinguish those RN patients who are at risk of large RN volume and poor QOL. Patients with high ApoB/ApoA1 ratio could be followed more closely, with the potential to identify large RN volume earlier. Hence, elevated ApoB/ApoA1 ratio can inform the clinician about the impact of lipoprotein on RN volume and QOL, which can be used to guide clinical management.

There are several limitations of our findings. First, cross‐sectional analysis may identify correlations between variables but no causality, which do not describe how serum lipids, lipoproteins, and ApoB/ApoA1 ratio, WHOQOL‐BREF scores in subjects with RN vary over time. Second, similar to many previously studies, we did not demonstrate differences in diet or exercise levels among study groups. Despite these limitations, as for the RN population, we included a relatively large number of patients. More importantly, we demonstrated that ApoB/ApoA1 ratio was an independent risk factor for RN volume and QOL. The clinical implications of our present findings cannot be ignored. First, our results implied that lipid metabolism may be involved in the development of RN. Therefore, interventions or medications targeted at lowing the ApoB/ApoA1 ratio may be a potentially effective strategy in prevention of RN and improvement of QOL. Finally, with the exploratory aim of this study, we hope to see more future studies investigating the underlying causal relationships among ApoB/ApoA1 ratio, RN, and QOL, with a particular focus on longitudinal assessment of postradiation ApoB/ApoA1 ratio.

## 5 CONCLUSION

In summary, we observed that elevated serum ApoB/ApoA1 ratio is positively correlated with RN volume and negatively correlated with QOL, which indicated serum ApoB/ApoA1 ratio as an independent risk factor for RN volume and QOL in RN patients after radiotherapy for NPC, suggesting a bright intervention target in RN treatment. It may be beneficial for clinicians to conduct clinical trials on new therapeutic modalities in RN after radiotherapy for NPC with an elevated ApoB/ApoA1 ratio, and direct personalized treatment strategies.

## CONFLICT OF INTEREST

None declared.

## Data Availability

The data that support the findings of this study are available from the corresponding author upon reasonable request.
